# Challenges of Cross-Sectoral Video Consultation in Cancer Care on Patients’ Perceived Coordination: Randomized Controlled Trial

**DOI:** 10.2196/60158

**Published:** 2025-02-11

**Authors:** Fereshteh Baygi, Theis Bitz Trabjerg, Lars Henrik Jensen, Maria Munch Storsveen, Sonja Wehberg, Jeffrey James Sisler, Jens Søndergaard, Dorte Gilså Hansen

**Affiliations:** 1 Department of Public Health, Research Unit of General Practice, University of Southern Denmark, Campusvej 55, Odense, 5230, Denmark, 45 65502348; 2 Department of Oncology, Lillebælt University Hospital, Vejle, Denmark; 3 The Department of Regional Health Research, Danish Colorectal Cancer Center South, Center of Clinical Excellence, Vejle Hospital, University of Southern Denmark, Vejle, Denmark; 4 Department of Family Medicine, Faculty of Health Sciences, University of Manitoba, , Winnipeg, MB, Canada, Canada

**Keywords:** randomized controlled trials, video consultations, outcome assessment, patients’ satisfaction, patients’ care coordination, interprofessional relations, cancer

## Abstract

**Background:**

Patients with cancer need coordinated care for both treatment and concurrent health conditions. This requires collaboration among specialists when using telemedicine services, emphasizing the importance of care continuity.

**Objective:**

This study aimed to explore the effects of cross-sectorial video consultation involving oncologists, general practitioners, and patients with cancer on patients’ perceived coordination of care, compared with usual care.

**Methods:**

This study describes the primary outcomes from a 7-month follow-up of patients in the Partnership Project, a randomized clinical trial. Patients in the intervention group were randomized to receive a “partnership consultation,” a shared video consultation with an oncologist, general practitioners, and the patient, in addition to their usual care. Questionnaires were completed for both groups at baseline and 7 months to assess the primary outcome, “global assessment of inter-sectorial cooperation,” from the Danish questionnaire “Patients’ attitude to the health care service.” The questionnaire also included 2 single items and 5 index scales, examining patients’ attitude toward cooperation in the health care system. Change in perceived global coordination from baseline to 7 months was compared between intention-to-treat groups using generalized estimating equations in a linear regression model.

**Results:**

A total of 278 participants were randomized with 1:1 allocation, with 80 patients receiving the intervention. Further, 210 patients completed the questionnaire at baseline, while 118 responded at 7-month follow-up. The estimated difference in the primary outcome between usual care (−0.13, 95% CI −0.38 to 0.12) and intervention (0.11, 95% CI −0.11 to 0.34) was 0.24 (95% CI −0.09 to 0.58) and not statistically significant (*P*=.15).

**Conclusions:**

Low rates of intervention completion and high levels of missing data compromised the interpretability of our study. While we observed a high level of global assessment of coordination, the estimated intervention effect was smaller than anticipated, with no significant difference in perceived coordination between control and intervention groups. Future studies should explore strategies like patient incentives to increase response rate and improve the evaluation of this innovative health care model.

## Introduction

Health care systems increasingly use digital technology to improve quality of health care services across a spectrum of medical issues including critical conditions like cancer [1]. Notably, over the past 2 decades, there has been a growing deployment of telemedicine technologies, making a transformative shift in how health care is delivered and experienced [2].

Patients with cancer have distinctive medical requirements, including both cancer therapy and treatment of concurrent health conditions [3]. Addressing these needs involves engaging various specialties and health professionals, using specialized telemedicine care services, and ensuring continuity of care throughout and after cancer treatment. This necessitates a higher level of care coordination [4]. However, both patients and health care providers face challenges in coordinating care and communication patterns, as evidence by logistic issues such as technological problems which hinder effective telemedicine practices [5,6]. To mitigate such challenges, shared care models have been proposed as a promising approach [7]. These models allow patients to benefit from the expertise of specialists, while maintaining the care through the primary care providers. By bringing together the patients, general practitioners (GPs), and oncologist in a shared video consultation, telemedicine offers a powerful solution to improve care coordination. A recent study further supports the benefits of telemedicine as its ability to improve care coordination, and better management patients’ health needs through enhanced communication [8]. Therefore, efforts to assist patients with cancer have shifted toward patient-centered communication approaches [6], so that over time, such approaches for these patients are rapidly expanding and diversifying [9]. Based on a previous study, these approaches may have varying impact on patients’ outcomes and perceptions [9]. Furthermore, application of such approaches in combination with virtual consultations may have diverse effects on outcomes, as they may interact differently with each patient’s unique health needs. This aligns with the health care providers’ perspective, who advocate patient-centered approaches in cancer care [9]. Despite such widespread advocacy, there is limited consensus on definition and methods to achieve patient-centered care [9].

A previous meta-analysis on cancer care coordination suggests that implementation of cancer-care coordination approaches resulted in positive changes in majority of measured outcomes (eg, overall patients’ experience on cancer care, quality of end-of-life care, etc). The study recommended the development of new intervention models and care coordination strategies to enhance patients’ self-management [10]. Notably, none of the studies included in this meta-analysis applied a virtual intervention mode [10].

We hypothesized that virtual shared models involving specialists, primary care providers, and patients could more effectively address optimal outcomes for patients with cancer [3]. Hence, this study aims to investigate the effects of a shared video consultation including oncologists, GPs, and patients with cancer on the patients’ perceived coordination of care.

## Methods

### Study Design

This study is a randomized controlled trial entitled “The Partnership Project’ (PSP)” [11]. The protocol and details of the study have been published previously [11-13]. This paper now presents the primary outcome based on a 7-month follow-up survey on patients’ participation in a shared video-based consultation.

### Participants and Setting

All newly diagnosed patients with any type of cancer receiving treatment with chemotherapy for the first time at the Department of Oncology, Lillebælt Hospital, University Hospital of Southern Denmark were invited for the study. The eligibility criteria were age above 18 years, proficiency in speaking and reading Danish, and having an estimation from an oncologist indicating a survival time of more than 7 months.

Multimedia Appendix 1 provides an explanation of the initial sample size that was previously published [14]. Since patient inclusion matched the predetermined sample size, the trial was ended.

### Usual Care

The control group was randomized to receive “usual care” in terms of standard information exchanging between the department of oncology and primary care. This involved sending an electronic summary letter to the GP after each visit to the department of oncology. GPs and the hospital can communicate by phone if needed. In addition, patients may reach out to their GP or a designated coordinator at the department of oncology.

### Intervention

Patients in the intervention group were randomized to receive a “partnership consultation,” which was a shared video consultation involving an oncologist, GP, and patients with cancer, alongside their “usual care.” GPs were contacted only after obtaining patients’ consent, and the GP had the option to refuse participation. Three to 6 weeks in advance, the sessions were scheduled during regular clinic hours. Patients were given the option to choose either the GPs’ office or the oncologist’s office for their consultations. In case the patient preferred to sit by the GP, the video consultation took place in that way, with the oncologist alone in his or her office at the hospital. GPs or oncologists may have had more than 1 patient in the intervention group. However, we do not have specific information about the individual oncologist for each patient in our database. The consultations were chaired by an oncologist within 12 weeks from the time of inclusion. Before the consultation, oncologists and GPs received information including a consultation guide with themes that may be relevant (Multimedia Appendix 2). Typically, the oncologist was assisted by an oncology nurse. A summary of the consultation was recorded in the hospital electronic patient record, shared with the GP, and accessible for the patient at an online portal (sundhed) for medical reports in Denmark.

Three to 6 weeks in advance, the sessions were scheduled during regular clinic hours. Patients were given the option to choose either the GPs’ office or the oncologist’s office for their consultations. In case the patient preferred to sit by the GP, the video consultation took place in that way, with the oncologist alone in his or her office at the hospital.

GPs or oncologists may have had more than 1 patient in the intervention group. However, we do not have specific information about the individual oncologist for each patient in our database.

The consultations were chaired by an oncologist within 12 weeks from the time of inclusion.

Prior to the consultation, oncologists and GPs received information including a consultation guide with themes that may be relevant (Multimedia Appendix 2). Typically, the oncologist was assisted by an oncology nurse.

A summary of the consultation was recorded in the hospital electronic patient record, shared with the GP, and accessible for the patient at an online portal (sundhed.dk) for medical reports in Denmark.

### Randomization and Blinding

Following informed consent, patients were assigned in a 1:1 ratio through block randomization, with block sizes and sequences known only to the REDCap (Research Electronic Data Capture; Vanderbilt University) [15] data manager from our collaboration partners. The allocation was transparent for the patients, GPs, and oncologist. However, during baseline data collection, patients in the intervention group and enrolling nurse were kept blind of the randomization.

A project nurse at the research unit in the department of oncology conducted the randomization process and enrolled patients in the study following the patients’ consent. Neither patients nor their GPs and oncologists in the intervention group were blinded to the patients’ allocation status. Data analysts remained blinded to the allocation. GPs of patients in the control arm were not formally informed until they received the survey.

### Primary Outcomes and Instruments

Patients were asked to complete questionnaires at baseline, and after 4 and 7 months. Upon arrival at the department of oncology, patients received information, a consent form, and a paper-based baseline questionnaire, which outpatient nurse collected after enrollment. Follow-up questionnaires were sent electronically using REDCap [15], which securely managed distribution and response collection. However, patients could request paper-based follow-up with prepaid return envelope.

An overview of primary and secondary outcomes can be found in Multimedia Appendix 3. The primary outcome included the single item “global assessment of inter-sectorial cooperation,” which was part of the Danish questionnaire “Patients’ attitude to the health care service” [14]. The English questionnaire “The patient cancer diary” [16] served as the basis for the 26-item Danish questionnaire. The adaptation was done based on interviews with Danish cancer patients and caregivers [17] and the English questionnaire template [16]. The questionnaire was chosen because it measures the study’s aim, patients’ perceptions of cross-sector cooperation, and has previously been used in a Danish cancer study [14]. Single items were scored on a 5-point Likert scale from strongly agree (1) to strongly disagree (5). The “not relevant” category was coded as missing.

This questionnaire comprises also 2 other single items and 5 index scales (secondary outcome; Multimedia Appendix 3), examining patients’ attitude toward the cooperation in the health care system. There is no manual available for the questionnaire; however, 2 papers have been published that describe the validation and usage of the questionnaire [14,17]. For the 5 subscales, at most 1 missing was replaced by the mean of the other items in the corresponding subscale. A subscale was coded as missing if more than 1 single item in the scale were missing. The direction of the answer scale varied depending on the item. For instance, in the case of the primary outcome, a low score indicated a positive attitude toward the question, while for secondary outcomes (eg, global feeling of left in limbo), a high score indicated a positive attitude. However, for analysis purpose, all items were aligned so that a higher value indicated a positive attitude toward the questions. Primary and secondary outcomes were measured at the following time points: baseline, 4, and 7 months after baseline. Coding was done separately for each time point.

### Other Parameters

Demographic data for patients including age, gender, education, marital status, having child, work status, comorbidity, diagnosis or cancer type were assessed through questionnaire which was completed by patients at baseline.

### Deviation From the Protocol in Statistical Analyses

As outlined in the published protocol [11], the original statistical analyses plan aimed to conduct a simple group comparison at 7 months using *t* tests or Wilcoxon rank-sum tests. However, a deviation from the initial analysis strategy was decided due to the large amount of missing data associated with the primary variable at 7 months.

### Definition of Intervention and Control Groups for Analysis

The main analysis strategy followed the intention-to-treat (ITT) approach, defining groups by random allocation (control and intervention). As a second approach, we defined 2 as-treated grouping approaches. First, we split the intervention group by degree of intervention fidelity: 1 group had the intervention as defined by protocol, the second did not receive the intervention due to technical issues, while the third did not receive the intervention for other reasons. Then, as-treated group_1_ (AT1) comprised patients completing the video consultations; this group was compared with patients who did not receive the intervention (randomized to control or subgroup_1_ or subgroup_2_). As-treated group_2_ (AT2) comprised patients with planned video consultations, regardless of completion due to technical reasons (AT1 and subgroup_1_); this group was compared with patients randomized to controls and subgroup_2_. As-treated groups were defined post hoc. Unless stated explicitly otherwise, we based the group definition on the ITT approach.

### Revised Statistical Analysis

As a first step, we compared the change from baseline to 7 months between the 2 groups, as defined by allocation (ITT), through a linear regression model. This model was applied to measurements at both baseline and 7 months, using generalized estimating equations (GEE) to account for within-patient clusters. Robust variance estimation was used, and the group difference was modeled as time-by-group interaction. Similar estimates were presented for both post hoc and defined as-treated approaches. No additional covariates were considered in this primary analysis. The GEE approach was chosen to ensure robustness of the statistical methods in the presence of missing data. Analyses specified in the protocol were also reported, restricted to complete cases. A corresponding analysis strategy was followed for secondary outcomes. Data analyses was done using Stata version 17 [18], and the significance level was set at 5%.

### Ethical Considerations

#### Statement Regarding Human Subject Research Ethics Review

The Regional Ethics Committee on Biomedical Research in Denmark (S-20142000‐138) and the Danish Data Protection Agency (2014-41-3534) peer reviewed and approved the study.

#### Informed Consent Descriptions

At the Oncological Department, Vejle Hospital, Denmark, outpatient clinic nurses obtained informed consent from patients for the PSP. Patients provided consent to participate in the randomized controlled trial, the video recordings, and the user perspective assessments on the same consent form. The consent forms were securely stored at the Clinical Research Unit, Department of Oncology, Vejle Hospital. The unit of randomization was the patient. Therefore, according to Danish law and the instructions of the Regional Ethics Committee on Biomedical Research, consent from GPs was not required. However, out of courtesy and to show consideration for their workload, oral consent was obtained from GPs when their patients were allocated to the intervention group. Before the study’s start, written information about the trial was sent to all GPs in the Region of Southern Denmark. If a GP declined to participate, their patients were not invited to join the study.

GPs for patients in the control group were not contacted and were therefore unaware of their patients’ participation in the study until they were asked to complete a questionnaire 4 months after the patients’ inclusion.

#### Privacy and Confidentiality Protection Description

Data security in video consultations is essential. Patients demonstrate a high level of trust regarding data security, as they trust the health care staff using the technology. To ensure this trust, all video consultations were conducted on the Region of Southern Denmark’s secure videoconference servers using virtual meeting rooms. These servers provide a highly secure connection with no third-party data processing, and meeting rooms could only be accessed by the participating parties. Before a video consultation, patients may have discussed confidential matters, such as alcohol consumption or smoking, which they had not shared with all health care providers. This could place patients in a dilemma. To address this, the intervention guide for oncologists and GPs includes a note to handle such situations sensitively.

#### Compensation Details

No compensation was provided to patients or oncologists. GPs were reimbursed through the standard payment system of the Region of Southern Denmark for participating in video consultations with a specialist at the hospital. The agreement used existing provisions for cross-sector cooperation and discharge follow-up. GPs received a fee for video consultations based on the time spent: €48 (at the time of the study, the exchange rate was approximately €1=US $1.10; fee number 4670) for up to 30 minutes and €97 (fee number 4669) for consultations exceeding 30 minutes. Therefore, they did not receive any additional payment for participation, nor were they paid by the study for their involvement in the video consultations. Furthermore, GPs were compensated for completing the questionnaires. Payment was provided by the Region of Southern Denmark and corresponded to one module (€18 at the time for the trial), equivalent to the payment for a standard patient consultation in their clinic.

## Results

### Recruitment and Participant Flow

**Figure 1.**
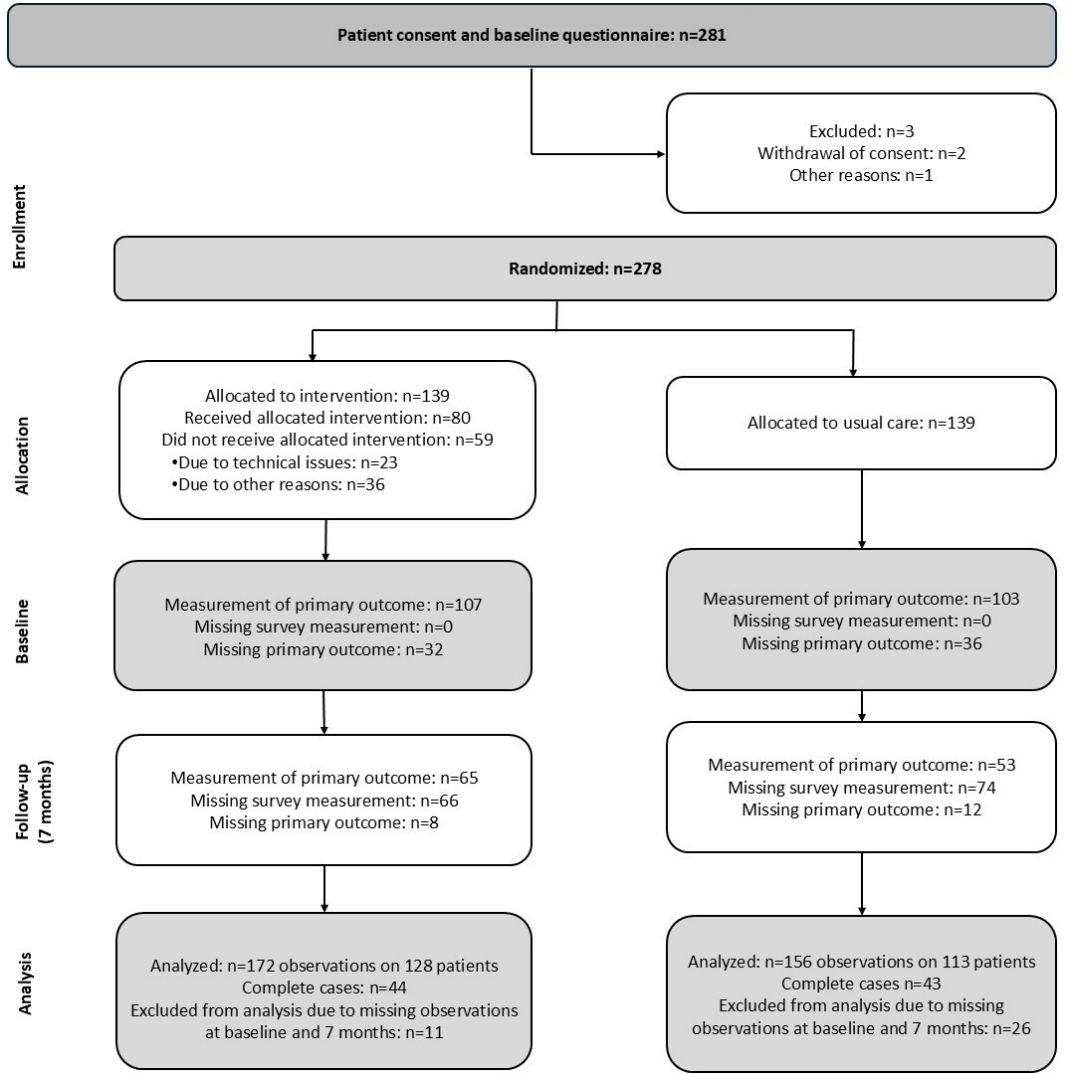
CONSORT (Consolidated Standards of Reporting Trials) flow diagram.

The patients were included between June 2016 and November 2019. In this study, 281 patients initially agreed to participate. Three patients were excluded due to withdrawal of the consent or other reasons. In total, 278 patients were randomized; 139 patients were allocated to the intervention group and another 139 to the control group. However, due to the following reasons, only 80 patients received the intervention as intended: GP refused participation for 22 patients; in 15 cases, IT failed; and for 8 patients, there were administrative (scheduling) issues. A total of 8 patients died before intervention, 3 patients were too ill, and 3 did not wish to participate in the intervention. See Figure 1 for the CONSORT (Consolidated Standards of Reporting Trials) flow diagram [19] (Checklist 1).

An overview of the GP-patient relationship can be found in Multimedia Appendix 4 to provide additional context.

### Baseline Data

As shown in Table 1, patients in the intervention and control groups had similar baseline characteristics. However, comorbidity was more frequent in the control group (58.3% vs 46.8%) than in the intervention group.

**Table 1.** Baseline characteristics of patients in the intervention and control groups.

Characteristics		Total (N=278), n (%)	Control group (n=139), n (%)	Intervention group (n=139), n (%)
Age (years), mean (SD)		65.2 (10.6)	63.8 (11)	66.6 (10)
Sex				
	Male	155 (55.6)	77 (55.4)	78 (56.1)
	Female	123 (44.4)	62 (44.6)	61 (43.9)
Education				
	Primary and upper secondary school	176 (63.3)	85 (61.1)	88 (63.3)
	Further education	76 (27.3)	41 (29.5)	35 (25.2)
	Higher education	16 (5.8)	7 (5)	9 (6.5)
Marital status				
	Single or missing^a^	81 (29.1)	48 (34.5)	33 (23.7)
	Married or residing with a companion	197 (70.9)	91 (65.5)	106 (76.3)
Children living at home				
	No children at home or missing^a^	244 (87.8)	120 (86.3)	124 (89.2)
	Children at home	34 (12.2)	19 (13.7)	15 (10.8)
Work status				
	Employed	89 (32)	46 (33.1)	43 (30.9)
	Public benefits	15 (5.4)	9 (6.5)	6 (4.3)
	Retired or missing^a^	174 (62.6)	84 (60.4)	90 (64.7)
Comorbidity				
	No	132 (47.5)	58 (41.7)	74 (53.2)
	Yes	146 (52.5)	81 (58.3)	65 (46.8)
Diagnosis or cancer type				
	Breast	33 (11.9)	17 (12.2)	16 (11.5)
	Gynecological	13 (4.7)	4 (2.9)	9 (6.5)
	Lung	106 (38.1)	53 (38.1)	53 (38.1)
	Gastrointestinal	110 (39.6)	56 (40.3)	54 (38.8)
	Other	16 (5.8)	9 (6.5)	7 (5)
	Incident cancer (yes or missing^a^)	255 (91.7)	126 (90.6)	129 (92.8)

a There were less than 3 patients with missing information on marital status, number of children at home or work status, and 6 patients with missing information on incident cancer. These patients were grouped with the indicated categories.

### Numbers Analyzed

In Table 2, an overview of the missing data for the primary outcome at different time points in both control and intervention groups is presented. Over time, there was a decline in participation in both the control group (38% at 7 months vs 74% at baseline) and the intervention group (47% at 7 months vs 77% at baseline), based on 278 randomized patients. In the ITT analyses, 172 observations on 128 patients from the intervention group and 156 observations on 113 patients from the control group were included. A total of 11 patients (intervention) and 26 patients (control) were excluded from the analysis due to missing observations at both baseline and 7 months.

A total of 59 participants failed to have the intervention as intended, due to technical problems or other reasons. Based on the subgroup analyses, the subgroup_1_ had a higher percentage (78.3%) of nonmissing values, which gradually dropped to 39.1%% and 34.8% at the subsequent time points (Table 2). The subgroup_2_ displayed a comparable pattern of missing data at various time points (83.8%, 35.1%, and 24.3%, respectively).

**Table 2.** Overview of the missing data for the primary outcome in both control and intervention groups at baseline and follow-up.

Group	Baseline					4 months				7 months			
	Total, n	Data available, n (%)	Data^a^ missing, n (%)	Not relevant^b^, n	Missing^c^ survey, n (%)	Data available, n (%)	Data missing, n (%)	Not relevant, n	Missing survey, n (%)	Data available, n (%)	Data missing, n (%)	Not relevant, n	Missing survey, n (%)
Total	278	210 (75.5)	68 (24.5)	51	0	138 (49.6)	27 (97)	14	113 (40.6)	118 (42.4)	20 (7.2)	0	140 (50.4)
Group_1_^d^	139	103 (74.1)	36 (25.9)	27	0	61 (43.9)	15 (10.8)	7	63 (45.3)	53 (38.1)	12 (8.6)	—^e^	74 (53.2)
Group_2_^f^	139	107 (77)	32 (23)	24	0	77 (55.4)	12 (8.6)	7	50 (36)	65 (46.8)	8 (5.8)	—	66 (47.5)
Subgroup_1_^g^	23	18 (78.3)	5 (21.7)	—	0	9 (39.1)	3 (13)	—	11 (47.8)	8 (34.8)	<3 (8.7)	—	13 (56.5)
Subgroup_2_^h^	36	31 (83.8)	6 (16.2)	—	0	13 (35.1)	2 (5.4)	—	22 (59.5)	9 (24.3)	<3	—	27 (73)

a Data missing: the primary variable was absent, while other sections might have been answered.

b Not relevant: the primary variable was answered as “not relevant”; this is part of the data missing category.

c Missing survey: the entire questionnaire was missing for a specific time point.

d Group_1_: randomized as control.

e Not applicable.

f Group_2_: randomized as intervention.

g Intervention group was divided into the following subgroups based on reasons for not receiving the intervention. Subgroup_1_: not received intervention due to technical problems.

h Subgroup_2_: not received intervention due to other problems.

### Outcomes and Estimations

Table 3 shows patients’ attitudes toward the cooperation between the primary sector and the department of oncology. The estimated within-group changes in the primary outcome between baseline and follow-up were −0.13 (95% CI –0.38 to 0.12) in the control group and 0.11 (95% CI −0.11 to 0.34) in the intervention group. The between-group difference was estimated as 0.24 (95% CI −0.09 to 0.58; *P*=.15). This suggest that, based on perceived global coordination, there was no noticeable differences between the ITT groups from the beginning to 7-month follow-up.

**Table 3.** Patients’ attitudes toward the cooperation between the primary sector and the department of oncology.

Outcomes			Group	Baseline		7 months		Estimated change (95% CI)	Group-time interaction,(95% CI)	*P* value
				n	Mean (SD)	n	Mean (SD)			
Primary										
	ITT^a^		C^b^	103	3.73 (0.98)	53	3.62 (1.04)	−0.13 (−0.38 to 0.12)	—^c^	—
	ITT		I^d^	107	3.79 (0.96)	65	3.91 (0.98)	0.11 (−0.11 to 0.34)	0.24 (−0.09 to 0.58)	.15
	AT1^e^		C	151	3.75 (0.96)	70	3.67 (1.09)	−0.10 (−0.33 to 0.12)	—	—
	AT1		I	59	3.78 (1)	48	3.94 (0.89)	0.17 (−0.08 to 0.42)	0.27 (−0.07 to 0.61)	.11
	AT2		C	133	3.71 (0.95)	62	3.65 (1.06)	−0.09 (-0.33 to 0.14)	—	—
	AT2		I	77	3.86 (1)	56	3.93 (0.95)	0.10 (−0.15 to 0.34)	0.19 (−0.15 to 0.53)	.27
Secondary (subscores)										
	LIMBO^f^									
		ITT	C	111	3.77 (1.18)	55	3.73 (1.18)	−0.05 (−0.39 to 0.29)	—	—
		ITT	I	112	3.96 (1.03)	64	3.81 (1.15)	−0.13 (−0.44 to 0.18)	−0.08 (−0.55 to 0.38)	.73
	FAM-Global^g^									
		ITT	C	117	12.79 (27.91)	42	6.12 (14.73)	−6.68 (-13.41 to 0.06)	—	—
		ITT	I	108	19.69 (35.65)	4	8.06 (19.41)	−11.65 (−20.44 to −2.87)	−4.97 (−16.04 to 6.09)	.38
	FAM-Information^h^									
		ITT	C	105	14.30 (2.02)	42	14.15 (2.22)	−0.29 (−0.99 to 0.42)	—	—
		ITT	I	94	14.16 (2.07)	45	14.38 (2.19)	0.20 (−0.48 to 0.87)	0.48 (−0.49 to 1.46)	.33
	FAM-Care^i^									
		ITT	C	108	20.6 (3.92)	42	20.19 (3.98)	−0.14 (−1.10 to 0.82)	—	—
		ITT	I	99	20.42 (3.92)	45	20.57 (4.57)	0.35 (−0.92 to 1.62)	0.48 (−1.11 to 2.08)	.55
	FAM-knowledge^j^									
		ITT	C	105	10.7 (2.83)	41	10.66 (2.74)	−0.19 (−0.83 to 0.45)	—	—
		ITT	I	95	11.28 (2.44)	46	11.89 (2.44)	0.71 (−0.04 to 1.46)	0.90 (−0.09 to 1.88)	.07
	LIMBO-Total									
		ITT	C	105	27.98 (5.59)	54	28.17 (5.34)	0.03 (−1.37 to 1.42)	—	—
		ITT	I	108	29.32 (4.81)	64	27.93 (5.63)	−1.58 (−2.95 to −0.21)	−1.61 (−3.56 to 0.35)	.11
	Coordination-Total									
		ITT	C	97	13.89 (3.50)	53	13.75 (3.45)	−0.15 (−1.12 to 0.81)	—	—
		ITT	I	106	14.29 (3.46)	61	15.19 (3.12)	0.90 (0.04 to 1.75)	1.05 (−0.23 to 2.34)	.11

a ITT: intention-to-treat approach.

b C: control.

c Not applicable.

d I: intervention.

e AT: as-treated groups (AT1 and AT2).

f LIMBO: global feeling of left in limbo.

g FAM-Global: global support from general practitioner.

h FAM-Information: information from general practitioner subscale.

i FAM-Care: support from general practitioner subscale.

j FAM-knowledge: general practitioners’ knowledge regarding treatment subscale.

In the context of the AT1 approach (Table 3), comparing patients who received the intervention with those who did not, the estimated within-group change in the primary outcome between baseline and follow-up was −0.10 (95% CI −0.33 to 0.12) in the control group and 0.17 (95% CI −0.08 to 0.42) in the AT1 group. The between-group difference was estimated as 0.27 (95% CI −0.07 to 0.61; *P*=.12).

For the AT2 approach (Table 3), the estimated within-group change in the primary outcome between baseline and follow-up was −0.09 (95% CI −0.33 to 0.14) in the control group and 0.10 (95% CI −0.15 to 0.34) in the AT2 group. The estimated between-group difference was 0.19 (95% CI −0.15 to 0.53) with the corresponding *P=*.27.

The estimated within-group and between-group changes in all secondary outcomes including 2 single items and 5 subscales showed no significant differences between the ITT groups from the beginning to 7-month follow-up (Table 3).

We also conducted the originally specified analyses on the primary outcomes at 7 months only, comparing intervention and control group in the ITT, AT1, and AT2 approach. The findings are presented in Table 4, showing similar *P* values.

**Table 4.** Additional analyses on primary outcomes at 7 months.

	Difference of means at 7 months(IV^a^ minus control) (95% CI)	*P* value for *t* test	*P* value for Wilcoxon rank-sum test (exact)
ITT^b^	0.285 (−0.085 to 0.655)	.13	.11
AT1^c^	0.266 (−0.109 to 0.641)	.21	.21
AT2^d^	0.283 (−0.085 to 0.652)	.12	.12

a IV: intervention group.

b ITT: intention-to-treat approach.

c AT1: as-treated group_1_.

d AT2: as-treated group_2_.

## Discussion

### Principal Findings

This study found that the addition of a cross-sectoral video consultation to usual care did not significantly impact patients’ perceived coordination of care. Both intervention and control groups showed high levels of perceived coordination at both time points, with no statistically significant differences over time or between the groups.

### Comparison With Previous Work

Based on our knowledge, video consultation has been used for patients with cancer for many years [20]. A recently published systematic review showed virtual consultation over time has been developed and improved in many ways (eg, delivery platforms and stakeholder engagement) [21]. Despite this improvement, comparing our findings was challenging due to the lack of studies on patients’ attitudes toward care coordination in multidisciplinary video consultations, which involve patients with cancer, oncologists, and GPs simultaneously.

A newly released scoping review revealed that specialist collaborations with GPs and patients can increase the effective quality of care in the follow-up phase for patients with cancer [22]. This comprehensive review did not report findings on patients’ attitudes toward care coordination. Therefore, we believe that in this area, more studies should be initiated to capture a better picture of care coordination from patients’ perspective and subsequently enhance the quality-of-care coordination for patients with cancer.

### Limitations of the Study

Several limitations should be considered. First, a large percentage of participants in the intervention group did not proceed with the intervention, mostly due to GP refusal to participate, administrative or technical issues. This could affect the generalizability of our findings to the broader population or specific subgroups due to potential selection bias. However, it should be noted that the trial was carried out before the introduction of a standard, clinically available video setup during the COVID-19 pandemic. Since then, the technical aspects of video-based communication in everyday life and health care consultations have improved dramatically [23]. However, challenges related to establishing online meetings, achieving relevant views of all participants, and ensuring efficient sound quality persist. These issues can occasionally make scheduled consultations impracticable [24]. These facts highlight the relevance of the results of “the Partnership Study” and underscore the importance of our learnings for future health care, particularly in adapting the evolving landscape of telemedicine. Second, the low completion rate for video consultation (58%) and high rate of missing data in our study affected the quality of our data, consequently limited our ability to draw definitive conclusion on effectiveness of the intervention. As a result, we focused on addressing challenges encountered. Third, a considerable amount of missing data for the primary outcome at the 7-month time point might impact the statistical power and consequently lead to a lack of significance in our findings. Several factors can be contributed to this issue (eg, the lengthy follow-up period, focus of the process evaluation on the intervention rather than barriers to participants retention, inadequate assessment of the follow-up duration during the pilot test, reliance on survey distribution alone, particularly during COVID-19 pandemic). In addition, it is possible that a ceiling effect influenced the intervention’s lack of superiority, as coordination scores were substantially higher, compared with findings from another department [14]. Fourth, the choice of single primary outcome variable that included “not relevant” response option may have constrained the depth of data obtained, because patients who had not experienced collaboration might have selected “not relevant.” Fifth, in the as-treated analysis, some patients from the intervention group were combined with patients from the control group, which assumes that the nonreceipt of the intervention was unrelated to individual patients’ characteristics. Therefore, our findings should be interpreted with caution. Sixth, the study does not provide insight into why some patients marked the primary outcome as “not relevant.” It is possible that these patients had no experience with collaboration at the time of the survey, and this uncertainty limits our understanding of the factors contributing to missing data and how they may influence the study’s conclusions. The handling of responses marked as “not relevant” as missing data may not actually capture patients’ experiences, that raise concerns about the interpretation of the results (eg, generalizability of findings related to collaboration experiences).

### Future Direction

Despite the lack of significant differences on primary and secondary outcomes of care coordination, limitations of our study may have implications for the research community in their future studies. For instance, our findings stress the need for further exploration into structural and engagement factors to strengthen future interventions.

The low completion rate for video consultation may indicate logistical and engagement challenges. Therefore, we encourage researchers in their future similar intervention to implement strategies that enhance patients’ engagement and improve data completion rate.

We encountered challenges such as potential power problem in reaching statistical significance. Despite this, we believe it is crucial to delve deeper into these findings and explore underling factors. This could provide valuable insights for development of more effective interventions in future. Findings may also highlight more involvement of patients to address their concerns related to care coordination and consequently enhance their experiences with the health care system. Furthermore, the findings highlight the importance of continually evaluation of health interventions to understand the impacts over time and make timely and necessary improvement, particularly where we have clearly identified specific problems or challenges, like decreased patients’ satisfaction.

### Conclusion

Our study, conducted before the COVID-19 pandemic, found that the shared video consultation model, involving patients with cancer, oncologists, and GPs, did not result in a statistically significant difference in patients’ perceived coordination of care between the control and intervention groups. We suggest that technical issues impacting the intervention implementation and the potential ceiling effect may have contributed to these results. Therefore, we emphasize the necessity for additional evaluation of the conceptual notion of uniting patients with cancer, oncologists, and GPs, particularly considering the advancements in techniques, the adoption of virtual communication, and the expanding role of GPs in cancer care. Further exploration of specific aspects of care coordination may provide additional insights into areas for improvement in this innovative health care model. This study highlights the complexity of implementing collaborative health care interventions and emphasizes the importance of ongoing evaluation of the intervention to optimize patients’ care coordination in cancer management. In addition, future research should focus on evolving trends in virtual communication among professionals and the public, as we think that leveraging post-COVID virtual communications could improve future health care interventions.

## Supplementary material

10.2196/60158 Multimedia Appendix 1 Original sample size.

10.2196/60158 Multimedia Appendix 2 The consultation guide for general practitioners and oncologists, including themes potentially relevant for the consultation.

10.2196/60158 Multimedia Appendix 3 Overview of primary and secondary outcomes.

10.2196/60158 Multimedia Appendix 4 Overview of general practitioner–patient relation.

10.2196/60158 Checklist 1 CONSORT-EHEALTH checklist (V 1.6.1).

## Abbreviations:

AT1: as-treated group_1_
AT2: as-treated group_2_
CONSORT: Consolidated Standards of Reporting Trials
GEE: generalized estimating equations
GP: general practitioner
ITT: intention-to-treat
PSP: Partnership Project
REDCap: Research Electronic Data Capture
