# Supplementary material for: Challenges of Cross-Sectoral Video Consultation in Cancer Care on Patients’ Perceived Coordination: Randomized Controlled Trial
Source: JMIR Cancer. 2025 Feb 11;11:e60158. doi: 10.2196/60158 (PMC11835449; doi:10.2196/60158)
Supplement: Multimedia Appendix 1 [file cancer-v11-e60158-s001.docx]

**Multimedia Appendix 1:**

***Original Sample size***

We based the sample size calculations on a Danish RCT evaluating an intervention aiming to improve the role of the GP in cancer follow up- by using this outcome [15]. In that study, mean values were reported as 56.6 for the control group and 69.6 for the intervention group, with a common standard deviation of 27. By using these estimates and a clinically significant difference of 20% between groups, we determined that a total sample size of 194 (2 × 97) would be necessary, with a significant level of 0.05 for two- sided tests and a 10% risk of type II error, i.e. a power of 90%. However, expecting a dropout rate of 30%, our recruitment goal is to enroll 278 patients.
