# Supplementary material for: Challenges of Cross-Sectoral Video Consultation in Cancer Care on Patients’ Perceived Coordination: Randomized Controlled Trial
Source: JMIR Cancer. 2025 Feb 11;11:e60158. doi: 10.2196/60158 (PMC11835449; doi:10.2196/60158)
Supplement: Multimedia Appendix 2 [file cancer-v11-e60158-s002.docx]

**Multimedia Appendix 2:** The consultation guide to GPs and oncologists, including themes potentially relevant for the consultation.

| **Consultation guide:** |
| --- |
| - The oncologist acts as chair of the shared video consultation - The duration of the consultation should be between 10 to 20 minutes - The oncologist starts by introducing the participants and the purpose of the shared consultation - Exchange of information between all participants for the benefit of the patient - Role and tasks clarification between the Department of Oncology and the GP - The consultations conclude with a summary, in which it is clarified whether a follow-up is needed with the GP or Department of Oncology - The consultation and its agreements are documented in the hospitals' electronic patient record and sent to the GP, and made available for the patient online |
| **The list of potential themes (not all themes might be relevant for the patient):** |
| - A summary of the patient trajectory - Patients concerns and desire for the consultation - Sharing knowledge regarding comorbidity - Psychosocial resources and needs - Agreements on who should take care of what and when in the future - Physical and psychological well-being - Medicine - Relatives - Ability to work - Late complications and side effects to the treatment |
