# Supplementary material for: Challenges of Cross-Sectoral Video Consultation in Cancer Care on Patients’ Perceived Coordination: Randomized Controlled Trial
Source: JMIR Cancer. 2025 Feb 11;11:e60158. doi: 10.2196/60158 (PMC11835449; doi:10.2196/60158)
Supplement: Multimedia Appendix 3 [file cancer-v11-e60158-s003.docx]

**Multimedia Appendix 3:** Overview of primary and secondary outcomes

| **Variable** | **Short name** | **Item number** | **Reversed** |
| --- | --- | --- | --- |
| Primary variable: global coordination |  | n=1: 1 | 1 |
| **Secondary variables: single variables** |  |  |  |
| Global feeling of left in limbo |  | n=1: 2 | - |
| Global Support from general practitioner |  | n=1: 3 | - |
| **Secondary variables: subscales** |  |  |  |
| Coordination subscale | Coordination | n=4: 4-7 | 5, 7 |
| Feeling left in limbo subscale | LIMBO | n=7: 8-14 | 9, 14 |
| Support for general practitioner subscale | FAM-Care | n=5: 15-19 | 15-19 |
| Information from general practitioner subscale | FAM-Information | n=4: 20-23 | - |
| General practitioners’ knowledge regarding treatment subscale | FAM-Knowledge | n=3: 24-26 | 24-26 |
