# Supplementary material for: Challenges of Cross-Sectoral Video Consultation in Cancer Care on Patients’ Perceived Coordination: Randomized Controlled Trial
Source: JMIR Cancer. 2025 Feb 11;11:e60158. doi: 10.2196/60158 (PMC11835449; doi:10.2196/60158)
Supplement: Multimedia Appendix 4 [file cancer-v11-e60158-s004.docx]

**Multimedia Appendix 4:** Overview of GP-patient relation

| GPs | All (n) | Only control | Only intervention | Both groups |
| --- | --- | --- | --- | --- |
| 1 patient | 152 | 68 | 84 | - |
| 2 patients | 38 | 14 | 5 | 19 |
| 3 patients | 8 | - | 2 | 6 |
| 4 or 5 patients | 6 | 1 | - | 5 |
